# Factors associated with adults’ actions to confirm their own rubella immune status in Japan’s drive toward rubella elimination: Cross-sectional online survey of non-healthcare workers in their 20s to 40s

**DOI:** 10.1186/s12199-021-01002-7

**Published:** 2021-08-11

**Authors:** Masataro Norizuki, Ai Hori, Koji Wada

**Affiliations:** 1 Graduate School of Medicine, International University of Health and Welfare Graduate School, 4-1-26 Akasaka, Minato, Tokyo, 107-8402 Japan; 2 Bureau of International Health Cooperation, National Center for Global Health and Medicine, 1-21-1 Toyama, Shinjuku, Tokyo, 162-8655 Japan; 3 Department of Global Public Health, University of Tsukuba, 1-1-1 Tennodai, Tsukuba, 305-8577 Japan

**Keywords:** Rubella, Vaccine-preventable disease, Knowledge, Attitude, Immunization policy, Supplementary immunization activities

## Abstract

**Background:**

Rubella outbreaks occurred among adults in Japan in 2013-2014 and 2018-2019 due to immunity gaps. In response and aiming at rubella elimination by 2020, the government introduced countermeasures comprising supplementary immunization activities for voluntary testing of adult non-healthcare-related workers and vaccination of susceptible individuals. However, as of October 2020, rubella immunity testing and vaccination rates remained low. This study was conducted to identify factors associated with adults voluntarily confirming their rubella immune status, to help develop effective promotion activities for hard-to-reach and left-behind populations.

**Methods:**

In this cross-sectional study, a general population sample of non-healthcare workers aged 20-49 years in Japan completed an online survey in November 2020. Univariate analysis was performed to examine associations of specific actions taken to confirm rubella immune status with social background characteristics, knowledge of rubella, and attitude to testing and vaccination. Log binomial regression analysis was performed to explore the associations following adjustment for social background characteristics.

**Results:**

Among 1,854 respondents (927 men, 927 women), only 23.4% of men and 39.4% of women in their 20s to 40s have taken some action related to rubella prevention. Three major factors were associated with the targeted population having taken voluntary action: (1) knowing about testing for confirmation of immunity status (adjusted odds ratio [AOR] 4.29 men, 2.89 women), the rubella outbreak in 2013 among men in their 20s to 40s (AOR 2.79 men, 1.64 women), and congenital rubella syndrome (AOR 1.89 men, 3.10 women); (2) having acquaintances who were vaccinated against or tested for rubella (AOR 2.98 men, 1.95 women); and (3) having a positive attitude toward influenza vaccination (AOR 2.48 men, 1.83 women). Marriage, desire for pregnancy, and having children were weakly associated with taking action.

**Conclusions:**

Currently, insufficient voluntary action is being taken by high-risk adult populations to close the identified immunity gaps. In this last mile to rubella elimination, our findings and suggested potential interventions via annual health check-ups and occupational health and public health initiatives could prove helpful in developing further countermeasures that actively promote and implement supplementary immunization activities targeting all adult generations.

## Background

Population immunity of at least 83% is needed to achieve herd immunity and interrupt transmission of endemic rubella virus [1]. Routine immunization with rubella-containing vaccine has dramatically decreased infections, and rubella has been successfully eliminated in some countries and regions [2]. However, even in some countries that have achieved high vaccination coverage in childhood, outbreaks have occurred among adults [3, 4]. In Japan in 2013, a rubella outbreak occurred among the working-age population that had not been previously vaccinated; 14,344 adults mostly in their 20s to 40s were infected and 45 cases of congenital rubella syndrome (CRS) were recorded [5–7].

This 2013 outbreak was due to immunity gaps. In 1977, the government had started a rubella vaccination program for adolescent females (age 12-15 years) to prevent CRS and the policy continued until 1995, when universal infant immunization was introduced. Vaccinations programs started in 1989 for the first dose of measles-rubella vaccine and in 2006 for the second dose [8]. The historical routine immunization of adolescent females, subsequent introduction of childhood immunization, and lack of supplementary immunization activities (SIAs) for testing of adults resulted in immunity gaps among adults born before April 1, 1990 who were not infected with rubella naturally and who did not have the opportunity to be vaccinated twice.

As a countermeasure to the 2013 outbreak, from 2014 the government introduced voluntary-based SIAs for adults. To promote vaccination uptake before pregnancy and prevent CRS cases, the government started providing financial support to women of childbearing age and their partners for antibody testing [9]. Despite these efforts, another outbreak occurred in 2018-2019 mainly among rubella-susceptible adult males [10], and 5 CRS cases were reported [11]. As an additional countermeasure and to try to meet Japan’s target of rubella elimination by 2020, in 2019 the government also began testing adult males for immunity and vaccinating males born between April 2, 1962 and April 1, 1979, who had no opportunity to receive routine vaccination against rubella and who were found by an antibody test to have a lower seroprevalence of rubella-specific antibody than other age groups [11, 12]. However, as of October 2020, among the targeted adult male population for example, only 16% ( 2,491,478/15,374,162) had been tested for rubella immunity (1,245,330 in 2019 and 1,246,148 in 2020) [12].

In countries close to eliminating rubella like Japan, vaccination needs to reach all susceptible adults. This requires that individuals confirm their own immune status for rubella, by checking their documented vaccination history or taking a rubella antibody test and getting vaccinated as necessary. However, because testing and vaccination strategies in Japan are voluntary, there is concern that SIA coverage is lower than needed to achieve herd immunity and eliminate rubella. Also, although far fewer rubella cases were reported in 2020 compared with previous years (100 in 2020 [13] vs 2,946 in 2018 [11] and 2,306 in 2019 [11]), social distancing, universal masking, and additional hygiene measures introduced during the novel coronavirus disease 2019 (COVID-19) pandemic likely contributed to this dramatic decrease, and another rubella outbreak may be possible when society opens up again.

We conducted this study in late 2020 to evaluate the latest progress in promoting high-risk adults to voluntarily confirm their rubella immune status. We investigated sociodemographic characteristics and attitudes associated with voluntary action in order to identify ways to help policy makers promote action among high-risk adults with lower immunity, especially those in their 20s to 40s and in hard-to reach or left-behind populations.

## Methods

### Participant enrollment

This cross-sectional study was conducted with a general population sample in Japan. Participants were recruited by an online survey company (Macromill, Inc. Tokyo, Japan). All registered candidates had agreed to participate in online surveys and could withdraw consent at any time, and additionally read the study information before specifically agreeing online to participate in this study, with the option to withdraw up until submitting their answers. Thus, written consent was not obtained. The institutional ethics committee approved this protocol.

Inclusion criteria were age 20-49 years and agreement to participate. Individuals were excluded if they were registered as healthcare-related workers according to 12 occupation categories and 43 subcategories used by the survey company (e.g., doctors, nurses, pharmacists, administrative staff working in hospitals, and employees of healthcare companies in order to better represent the general population, because healthcare workers tend to be vaccinated).

**Fig. 1:**
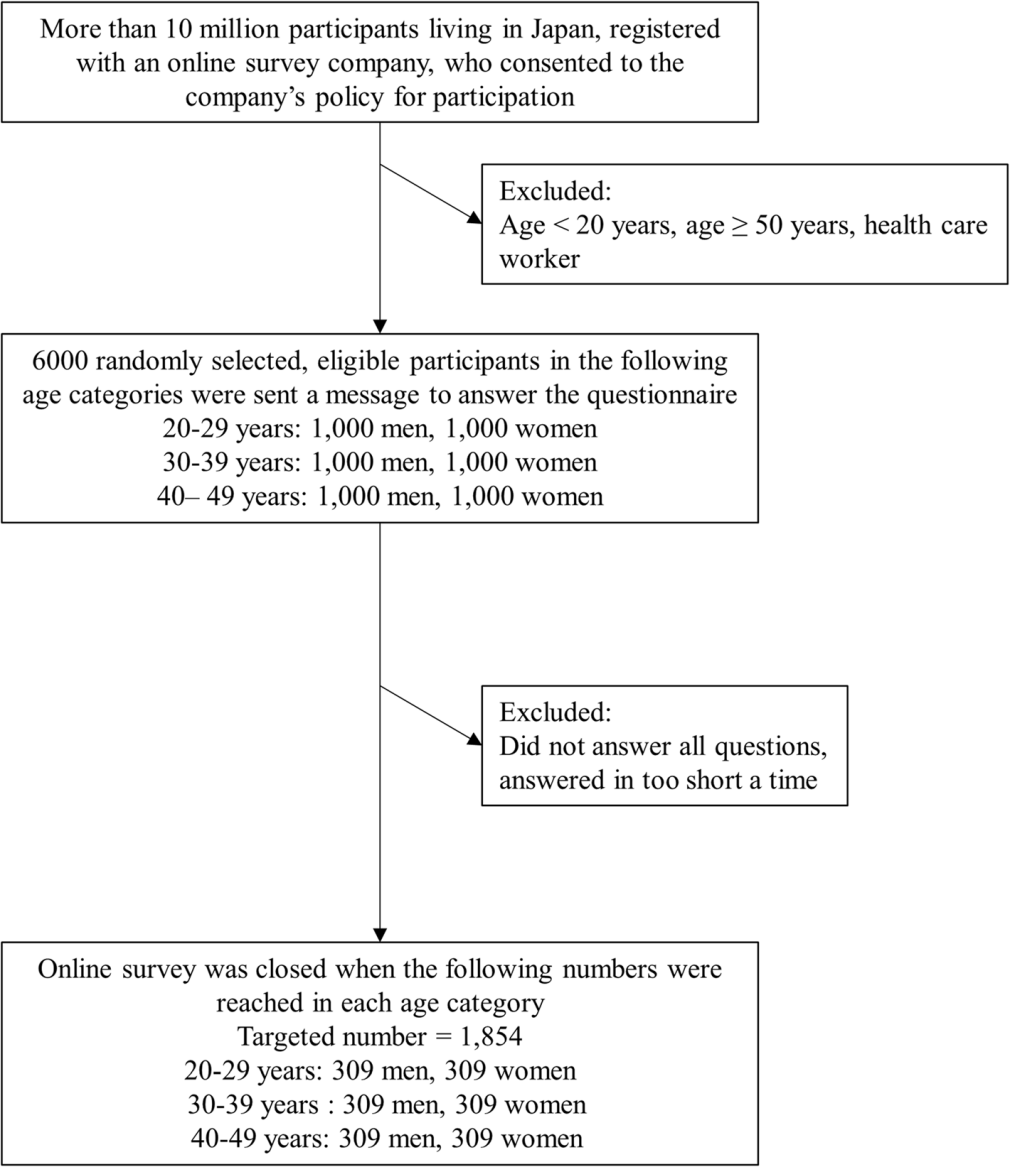
Participant flow in this cross-sectional online survey of non-healthcare workers in their 20s to 40s. Participants were enrolled from among non-healthcare workers aged 20 to 49 who had registered with an online survey company (Macromill, Inc. Tokyo, Japan). The survey company sent a message to 6,000 randomly selected registrants comprising 1,000 men and 1,000 women in three different age groups (20-29, 30-39, and 40-49 years). Recruitment was ended once approximately 1,800 participants agreed to participate, and ultimately 309 men and women in the three different age groups participated

In November 2020, from a list of 10 million individuals, the survey company randomly selected 6,000 eligible participants—1,000 men and 1,000 women in three different age groups (20-29, 30-39, and 40-49 years)—and invited them to participate. Registrants were provided financial incentives for their participation. Sample size was calculated based on a margin of error of 6%, assuming 50% of the target population in each stratum (3,500,000 men and 3,500,000 women), with 95% confidence intervals, to obtain 267 per stratum. We decided on a sample size of 300 for each of the six strata, and recruitment was ended once approximately 1,800 participants agreed to participate (Figure 1). Participants who did not answer all of the questions or answered in too short a time were excluded from the final analysis.

### Questionnaire data collection

#### Demographic information

The survey company provided basic demographic information on age, sex, marital status, and having children or not. Questionnaire items included additional basic demographic information, highest education level (high school or lower/junior college,/university/graduate school/no answer), and desire for pregnancy (“Are you, or your partner, pregnant or plan to get pregnant?” [Yes/No/I don’t know]).

#### Knowledge about rubella

To evaluate knowledge of rubella, based on current strategy and the government’s promotion for rubella elimination, we created three items on knowledge about the testing and vaccination strategy, the 2013 rubella outbreak, and CRS. Participants answered whether they knew or did not know the following information: “A blood test (antibody test) can be done to determine whether someone has been vaccinated against rubella or has previously had rubella” (I knew that/I did not know that); “In 2013, there was a rubella epidemic, mainly among men in their 20s to 40s” (I knew that/I did not know that); and “Women who are infected with rubella during pregnancy may give birth to children with congenital rubella syndrome. (Congenital rubella syndrome: difficulty hearing, heart deformities, sight problems, delayed mental or physical development, other congenital disorders)” (I knew that/I did not know that).

#### Attitude to testing and vaccination

We created two questions to reveal attitude toward testing and vaccination. To determine whether participants’ acquaintances had been vaccinated against rubella, the first question asked, “Since the 2013 epidemic, have any of your acquaintances (people you can contact by phone or email) had a rubella blood test (antibody test) or been vaccinated for rubella?” (Yes/No/I don’t know). To determine acceptance of vaccination, the second question asked, “Do you get an influenza vaccine?” (Every year/Almost every year/Sometimes/Never/I don’t know).

#### Actions to confirm rubella immune status

To identify whether respondents had taken action to confirm their rubella immune status, we asked the following three questions about action taken after the 2013 rubella outbreak in Japan: “Since the 2013 rubella epidemic, have you checked your Maternal and Child Health Handbook or other vaccination records to confirm whether you have been vaccinated for rubella?” (Yes/No/I don’t know); “Since the 2013 rubella epidemic, have you had a blood test (antibody test)?” (Yes/No/I don’t know); and “Since the 2013 rubella epidemic, have you had a rubella vaccination?” (Yes/No/I don’t know). We combined the answers to these three questions to generate a binary outcome variable; if respondents answered affirmatively to any of the three questions, we defined them as having taken action to confirm their rubella immune status.

### Statistical analysis

We performed univariate analysis using Pearson’s chi-squared test to examine the potential relationship between each of the three actions taken to confirm rubella immune status and social background, knowledge, or attitude. Log binomial regression analysis was then performed to explore the associations with adjustment for social background characteristics. All demographic variables were included in the primary model and all were shown to be significant in univariate analysis (P < 0.05) in this study or in previous studies [14–21]. The final model was adjusted for all variables. IBM SPSS Statistics 19 software (IBM Corporation, Armonk, NY) was used for all analysis.

### Ethical considerations

The study protocol was reviewed and approved by the Ethics Committee of the International University of Health and Welfare, Tokyo, Japan (approval number 18-Im-010).

## Results

### Participants

A total of 1,854 participants, 927 men and 927 women, participated in the web survey, which was conducted in mid-November 2020. Table 1 shows the participants’ sociodemographic characteristics and their reported actions to confirm their rubella immune status. More than 70% of female participants reported knowing about CRS, compared with less than half of male participants. More women than men had taken one or more of the three listed actions to confirm their rubella immune status (39.4% and 23.4%, respectively). Rubella antibody testing was the most common measure to confirm immune status among men and documented vaccination history was the most common measure to confirm immune status among women, with no significant difference found between the sexes.

**Table 1:** Sociodemographic characteristics of the 1,854 participants in this online survey (November 2020)

	Men		Women	
	n = 927	(%)	n = 927	(%)
**Having knowledge about**				
Confirmation of immune status by testing	259	(27.9)	428	(46.2)
2013 rubella outbreak in men in their 20s-40s	279	(30.1)	358	(38.6)
Congenital rubella syndrome	393	(42.4)	677	(73.0)
**Having acquaintances who got vaccinated or tested for rubella**				
Yes	94	(10.1)	147	(15.9)
No/ Don’t know	833	(89.9)	780	(84.1)
**Receiving influenza vaccination**				
Every year	184	(19.8)	185	(20.0)
Almost every year	134	(14.5)	143	(15.4)
Sometimes	223	(24.1)	234	(25.2)
Never	365	(39.4)	350	(37.8)
Don’t know	21	(2.3)	15	(1.6)
**Marital status**				
Married	342	(36.9)	492	(53.1)
Single	585	(63.1)	435	(46.9)
**Desire for pregnancy**				
Yes	260	(28.0)	306	(33.0)
No/ Don’t know	667	(72.0)	621	(67.0)
**Having a child/children**				
Yes	281	(30.3)	400	(43.1)
No/ Don’t know	646	(69.7)	527	(56.9)
**Age, years**				
20-29	309	(33.3)	309	(33.3)
30-39	309	(33.3)	309	(33.3)
40-49	309	(33.3)	309	(33.3)
**Highest education level**				
High school or lower	300	(32.4)	292	(31.5)
Junior college	117	(12.6)	258	(27.8)
University	431	(46.5)	342	(36.9)
Graduate school	59	(6.4)	26	(2.8)
No answer	20	(2.2)	9	(1.0)
**Taken voluntary action for rubella prevention after 2013 rubella outbreak**				
Any	217	(23.4)	365	(39.4)
Vaccination history*	112	(12.1)	257	(27.7)
Underwent antibody testing	129	(13.9)	209	(22.5)
Vaccination for rubella	106	(11.4)	148	(16.0)

*Confirmed more than two times from documented vaccination history, such as the Maternal and Child Health Handbook

### Binomial logistic regression analysis

Table 2 shows the prevalence of action taken to confirm immune status for rubella prevention stratified by sociodemographic background among men and women. Overall, 66.0% of men and 74.8% of women who had acquaintances who got vaccinated or tested for rubella took action to confirm own immune status for rubella. Among the different age groups, only 25.2% of women aged 40-49 years took action to confirm their rubella immune status.

**Table 2:** Prevalence of action for rubella prevention stratified by sociodemographic background among working-age men and women

	Men				Women			
	Action for rubella prevention		No action for rubella prevention		Action for rubella prevention		No action for rubella prevention	
	n = 217	(%)	n = 710	(%)	n = 365	(%)	n = 562	(%)
**Having knowledge about**								
Confirmation of immune status by testing	138	(53.1)	121	(46.7)	256	(59.8)	172	(40.2)
2013 rubella outbreak in men in their 20s-40s	131	(47.0)	148	(53.0)	191	(53.4)	167	(46.6)
Congenital rubella syndrome	160	(40.7)	233	(59.3)	328	(48.4)	349	(51.6)
**Having acquaintances who got vaccinated or tested for rubella**								
Yes	62	(66.0)	32	(34.0)	110	(74.8)	37	(25.2)
No/ Don’t know	155	(18.6)	678	(81.4)	255	(32.7)	525	(67.3)
**Receiving influenza vaccination**								
Every year	78	(42.4)	106	(57.6)	102	(55.1)	83	(44.9)
Almost every year	41	(30.6)	93	(69.4)	72	(50.3)	71	(49.7)
Sometimes	50	(22.4)	173	(77.6)	97	(41.5)	137	(58.5)
Never	46	(12.6)	319	(87.4)	92	(26.3)	258	(73.7)
Don’t know	2	(9.5)	19	(90.5)	2	(13.3)	13	(86.7)
**Marriage**								
Married	121	(35.4)	221	(64.6)	242	(49.2)	250	(50.8)
Single	96	(16.4)	489	(83.6)	123	(28.3)	312	(71.7)
**Desire for pregnancy**								
Yes	81	(31.2)	179	(68.8)	148	(48.4)	158	(51.6)
No/ Don’t know	136	(20.4)	531	(79.6)	217	(34.9)	404	(65.1)
**Having a child/children**								
Yes	108	(38.4)	173	(61.6)	211	(52.8)	189	(47.3)
No/Don’t know	109	(16.9)	537	(83.1)	154	(29.2)	373	(70.8)
**Age, years**								
20-29	64	(20.7)	245	(79.3)	124	(40.1)	185	(59.9)
30-39	64	(20.7)	245	(79.3)	163	(52.8)	146	(47.2)
40-49	89	(28.8)	220	(71.2)	78	(25.2)	231	(74.8)
**Highest education level**								
High school or lower	53	(17.7)	247	(82.3)	88	(30.1)	204	(69.9)
Junior college	26	(22.2)	91	(77.8)	106	(41.1)	152	(58.9)
University	119	(27.6)	312	(72.4)	153	(44.7)	189	(55.3)
Graduate school	17	(28.8)	42	(71.2)	16	(61.5)	10	(38.5)
No answer	2	(10.0)	18	(90.0)	2	(22.2)	7	(77.8)

Table 3 shows the associations found between sociodemographic factors and action taken to confirm rubella immune status among men and women, as determined by binomial logistic regression.

**Table 3:** Statistical associations between action for rubella prevention and sociodemographic background

	Men				Women			
	Crude OR	(95% CI)	Adjusted OR	(95% CI)	Crude OR	(95% CI)	Adjusted OR	(95% CI)
**Having knowledge about**								
Confirmation of immune status by testing	8.50	(6.06-11.93)	4.29	(2.88-6.38)	5.33	(4.00-7.10)	2.89	(2.00-4.18)
2013 rubella outbreak in men in their 20s-40s	5.78	(4.17-8.02)	2.79	(1.88-4.14)	2.60	(1.98-3.41)	1.64	(1.16-2.34)
Congenital rubella syndrome	5.75	(4.09-8.08)	1.89	(1.24-2.89)	5.41	(3.70-7.91)	3.10	(1.94-4.95)
**Having acquaintances who got vaccinated or tested for rubella**								
Yes	8.48	(5.35-13.44)	2.98	(1.70-5.23)	6.12	(4.10-9.14)	2.95	(1.84-4.71)
No/ Don’t know	Ref				Ref			
**Receiving influenza vaccination**								
Every year	3.20	(2.26-4.52)	2.48	(1.47-4.17)	2.24	(1.62-3.10)	1.83	(1.17-2.86)
Almost every year	1.55	(1.03-2.31)	1.49	(0.82-2.70)	1.70	(1.19-2.43)	1.53	(0.94-2.48)
Sometimes	0.93	(0.65-1.33)	1.55	(0.93-2.60)	1.12	(0.83-1.52)	1.58	(1.04-2.40)
Never	Ref				Ref			
Don’t know	0.38	(0.08-1.46)	0.68	(0.11-4.36)	0.23	(0.05-1.04)	1.60	(0.32-8.12)
**Marriage**								
Married	2.79	(2.04-3.80)	1.03	(0.56-1.91)	2.46	(1.87-3.23)	1.39	(0.93-2.06)
Single	Ref				Ref			
**Desire for pregnancy**								
Yes	1.77	(1.28-2.44)	1.54	(0.98-2.41)	1.74	(1.32-2.30)	1.39	(0.93-1.71)
No/Don’t know	Ref				Ref			
**Having a child/children**								
Yes	3.08	(2.24-4.22)	1.73	(0.93-3.22)	2.70	(2.06-3.55)	2.05	(1.28-3.30)
No/ Don’t know	Ref				Ref			
**Age, years**								
20-29	Ref				Ref			
30-39	0.79	(0.57-1.10)	0.83	(0.49-1.39)	2.30	(1.74-3.04)	1.11	(0.73-1.68)
40-49	1.55	(1.13-2.12)	1.25	(0.72-2.15)	0.39	(0.29-0.53)	0.17	(0.10-0.29)
**Highest education level**								
High school or lower	0.61	(0.43-0.86)	0.85	(0.54-1.33)	0.56	(0.42-0.75)	0.83	(0.56-1.24)
Junior college	0.93	(0.58-1.47)	1.06	(0.58-1.92)	1.10	(0.82-1.48)	1.11	(0.73-1.68)
University	Ref				Ref			
Graduate school	1.35	(0.75-2.43)	0.85	(0.40-1.82)	2.53	(1.14-5.64)	2.51	(0.94-6.68)
No answer	0.36	(0.08-1.55)	1.10	(0.20-6.20)	0.44	(0.09-2.12)	0.53	(0.80-3.44)

All listed variables were adjusted for in the final model.

OR, odds ratio; CI, confidence interval; Ref, reference

Men and women, but especially men, who knew that testing can confirm immune status were significantly more likely to have taken action to confirm their rubella immune status (men: adjusted odds ratio [AOR] 4.29, 95% confidence interval [CI] 6.06-11.93; women AOR 2.89, 95% CI 2.00-4.18). Similarly, both men and women who knew about the rubella outbreak in 2013 and about CRS were significantly more likely to have taken action to prevent rubella infection.

Men and women who took action to confirm their rubella immune status were more likely to have acquaintances who had received a rubella vaccination or had taken the antibody test (men: AOR 2.98, 95% CI 1.70-5.23; women: AOR 2.95, 95% CI 1.84-4.71). Both men and women who got an influenza vaccination every year were more likely to take action compared with those who never got an influenza vaccination (men: AOR 2.48, 95% CI 1.47-4.17; women: AOR 1.83, 95% CI 1.17-2.86).

Women with one or more children were significantly more likely to have taken action to confirm their rubella immune status (AOR 2.05, 95% CI 1.28-3.30), but only a weak association was seen among men (AOR 1.73, 95% CI 0.93-3.22).

Both being married and wanting to get pregnant tended to be associated with having taken action to confirm immune status in univariate analysis; however, the significance was weak after multivariate adjustment in men and women. Women aged 40-49 years were less likely to have taken action to confirm their rubella immune status compared with women aged 20-29 years (AOR 0.17, 95% CI 0.10-0.29), whereas men showed no significant difference between the two age groups (AOR 1.25, 95% CI 0.72-2.15). Highest education level showed no significant association with taking action.

## Discussion

This study revealed that, as of late 2020, there is currently insufficient voluntary action being taken by high-risk adults in the Japanese general population (excluding healthcare-related workers) to confirm their rubella immune status: only 23.4% of men and 39.4% of women in their 20s to 40s have taken some action related to rubella prevention. Thus, our findings indicate that there is still some way to go in achieving rubella elimination in Japan.

The first of the three major factors we found to be associated with the targeted population having taken voluntary action to check their rubella immune status was having knowledge about testing for confirmation of immunity status, about the rubella outbreak in 2013 among men in their 20s to 40s, and about CRS. It has been reported that knowledge about vaccine-preventable disease is closely associated with uptake of the influenza [15, 16], measles-rubella containing [22, 23], hepatitis A [24], and pertussis [25] vaccines. In the present study, knowledge that testing can confirm immune status showed a strong association with taking voluntary action among both men and women (men: AOR 4.29, 95%CI 6.06-11.93; women AOR 2.89, 95% CI 2.00-4.18). This suggests that Japan’s test and vaccination strategy has successfully prompted some of those reached by the promotion to actually take action. Further evidence that Japan’s current strategies are working are our findings that working-age men have become more aware of their role in CRS following the 2013 outbreak. An effective intervention to reach more of the 20- to 40-year-old target population could involve providing further information in the workplace about rubella the need to check immune status, and how to check it, via posters, pamphlets, or possibly occupational health activities inside larger companies.

The second factor that we found to be associated with taking voluntary action was having acquaintances who were vaccinated against or tested for rubella, and this association was found for both men and women. This is in agreement with the results of previous online surveys about rubella in Japan that reported the association for vaccination [14] and testing [17] of an acquaintance. Learning that a close acquaintance has been vaccinated or tested may instill a sense of security and highlight the benefits of taking action, which may be an effective strategy in collectivist societies like Japan [26]. Given that promotion activities through social networking sites such as Twitter and Facebook [27, 28] and through commercial and social marketing have been shown to be effective for vaccine uptake, activities involving familiar people might also be an effective strategy. Moreover, potential interventions could, for example, involve interested employees voluntarily sharing their experiences of vaccination during promotional activities run by occupational health personnel or encouraging people to share their experiences of testing or vaccination with friends through social networking service (SNS).

Lastly, among both men and women, getting vaccinated every year against influenza was associated with taking voluntary action to confirm rubella immunity, compared with never having been vaccinated against influenza. These findings are in agreement with other studies that have reported the association of annual influenza vaccination behavior with vaccine uptake [15, 16, 18]. People who have influenza vaccinations every year are likely to worry less about the side effects of the vaccine, be keenly interested in medical care, and have easy access to it. Therefore, it is important that promotions are targeted to reach people who are not interested or do not have easy access to medical care. A possible workplace-related intervention could be to include measurement and explanation of rubella antibody titer at the annual medical check-ups offered by companies in compliance with the labor law. This strategy could reach working-age company employees who are not interested in seeking out medical information, enabling them to easily confirm their immune status.

The life course events of marriage, desire for pregnancy, and having children were associated with taking voluntary action to confirm rubella status, albeit weakly in this study. Getting married tended to be associated with such action among men in the univariate analysis (OR 2.79, 95% CI 2.04-3.80) but not after multivariate adjustment (AOR 1.03, 95% CI 0.56-1.91), and men tended not to be interested in rubella before getting married but were more interested after getting married (AOR 1.03, 95% CI 0.56-1.91). An online study of the general adult population in 2014 also reported voluntary action was strongly associated with getting married [14]. We found that desire for pregnancy and having children showed weak associations with taking action, although the previous 2014 study found a strong association with desire to get married [14]. Also, a recent study conducted in 2020 with Japanese men aged 41-47 years found no association between partner’s current desire for pregnancy and undergoing rubella antibody testing (AOR 0.98, 95% CI 0.87-1.11) [17]. Because all pregnant woman are routinely tested for rubella antibody as part of antenatal care in Japan, experience of pregnancy is a confounder in women and is one of the reasons for the higher OR seen for women with children. Overall, our findings indicate that the switch from measures centered on pregnant women and their partners to measures centered on men in 2019 is having a positive impact, which is encouraging, but we need to reach more men to encourage them to take voluntary action to confirm their rubella immune status.

Surprisingly, we found that women in their 40s were significantly less likely to have taken voluntary action to confirm rubella immune status than those in their 20s. Current promotion activities mainly target pregnant women and men in their 40s, and the free testing and vaccination policy does not cover women without desire for pregnancy. Interest in rubella prevention has clearly fallen among women in their 40s. In addition, a recent report from the National Institute of Infectious Diseases indicates that seroprevalence is decreased among those aged 55-69 [29]. It is therefore necessary to design a system that allows all generations to confirm their own immunity with no one left behind.

We did not find an association between highest education level and taking action to confirm rubella immune status. There have been studies that have found an association between education level and vaccine intake—in the Bahamas [19], Lebanon [20], and United States [21]—and those that have found no such association—in Canada [30] and Japan [22]. The high literacy rate, easy access to written information, and high education level among all generations in Japan suggests that promotion activities can make good use of written information.

It seems that more men and women in their 20s to 40s have taken action as of late 2020 compared with 2013, when around 25% of women and men with desire for pregnancy but just 7.3% of women and 2.9% of men without a current desire for pregnancy had been tested for rubella antibody or vaccinated against rubella [14]. Even though our findings in late 2020 indicate that still only 39.4% of women and just 23.4% of men had taken some action to confirm their immunity, these are more encouraging rates, especially among women. Therefore, it seems then that the government’s testing and vaccination strategy that started targeting those with desire for pregnancy and their family members in 2019 is working to some extent [31] to close this identified immunity gap. This would also explain the gap in taking action between women and men in our study. It is important to note, however, that a recent survey in Japan reported that only 21.3% of men had actually been tested for rubella antibody [32]. So, while our study indicates that the government’s promotion activities have been reaching the targeted populations, there are still insufficient numbers of people taking the necessary preventive action to eliminate rubella in the very short term (originally 2020). Further strategies involving, for example, the annual medical check-ups, collaborations in occupational health activities, and encouragement to share experiences with friends through SNS are needed to promote action among those at high risk of rubella infection as well as hard-to-reach and left-behind populations.

Our study had some limitations. First, because data were obtained from a randomly selected online survey population, our findings cannot be considered representative of the general Japanese population as a whole. Second, self-report bias and selection bias may have resulted in overestimation of the results, although given that the targeted age group in their 20s to 40s tends to be familiar with information technology and to have internet access, we believe this did not adversely affect our primary objective of identifying factors associated with their participation in the SIAs. Third, this was a cross-sectional study, so we cannot infer causal relationships. Lastly, we did not consider the potential influence of the relationship between the rubella outbreaks and variation in efficacy of the live rubella vaccine across years. However, Japan does have strict quality control standards in place for vaccines [33]. Despite these limitations in study design, we believe that our up-to-date survey results are important to policy makers in deciding how to further accelerate measures for the elimination of rubella in Japan.

## Conclusion

Relying on voluntary action to confirm rubella immunity is currently not a sufficient strategy to reach herd immunity needed for rubella elimination. The main drivers for high-risk working-age adults to confirm their rubella immune status in Japan are (1) having knowledge about confirmation of immunity through testing, the 2013 rubella outbreak, and CRS, (2) having acquaintances who have had a rubella vaccination or test, and (3) having a positive attitude toward influenza vaccination. In this last mile to rubella elimination, the government should actively promote and implement SIAs to close the identified immunity gaps as well as provide additional ways for adults to easily check their rubella immune status and understand why it matters, such as via annual health check-ups and occupational health and public health initiatives, utilizing digital technology to help achieve this.

## Data Availability

The datasets used and analyzed during the current study are available from the corresponding author on reasonable request.

## Abbreviations

AOR: adjusted odds ratio
CI: confidence interval
COVID-19: novel coronavirus disease 2019
CRS: congenital rubella syndrome
OR: Odds ratio
Ref: Reference
SIAs: supplementary immunization activities
SNS: social networking service Social networking service
